# 4-Ethoxy­imino-*N*′-methoxy­pyrrolidin-1-ium-3-carboximidamidium dichloride

**DOI:** 10.1107/S1600536809004772

**Published:** 2009-02-21

**Authors:** Qiang Guo, Lanying Sun, Huiyuan Guo, Mingliang Liu

**Affiliations:** a Institute of Medicinal Biotechnology, Chinese Academy of Medical Sciences, and Peking Union Medical College, Beijing 100050, People’s Republic of China

## Abstract

The title compound, C_8_H_18_N_4_O_2_^2+^·2Cl^−^, contains two oxime groups. The methyl oxime group has a *Z* configuration, and the ethyl oxime group is disordered, with both *Z* and *E* configurations in occupancies of 0.840 (8) and 0.160 (8), respectively. In the crystal structure, there are a number of N—H⋯Cl hydrogen bonds.

## Related literature

For properties of quinolone derivatives, see: Ball *et al.* (1998 ▶); Ray *et al.* (2005 ▶). For the synthesis of new quinolones, see: Anderson & Osheroff (2001 ▶); Choi *et al.* (2004 ▶); Wang, Guo *et al.* (2008 ▶). For some crystal structures of quinolones, see: Wang, Liu *et al.* (2008 ▶). 
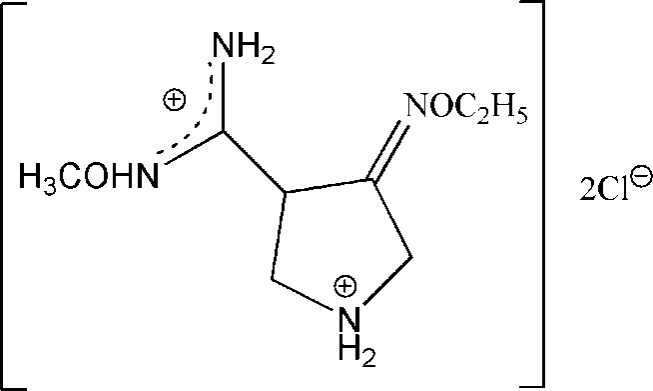


## Experimental

### 

#### Crystal data

C_8_H_18_N_4_O_2_^2+^·2Cl^−^
*M* *_r_* = 273.16
Orthorhombic, 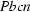

*a* = 12.7355 (14) Å
*b* = 8.8506 (12) Å
*c* = 26.334 (2) Å
*V* = 2968.3 (6) Å^3^
*Z* = 8
Mo *K*α radiation
μ = 0.43 mm^−1^
*T* = 298 K
0.23 × 0.20 × 0.19 mm

#### Data collection

Bruker SMART CCD area-detector diffractometer
Absorption correction: multi-scan (*SADABS*; Sheldrick, 1996 ▶) *T*_min_ = 0.907, *T*_max_ = 0.922
14370 measured reflections
2597 independent reflections
1986 reflections with *I* > 2σ(*I*)
*R*_int_ = 0.062

#### Refinement

*R*[*F* ^2^ > 2σ(*F* ^2^)] = 0.077
*wR*(*F* ^2^) = 0.210
*S* = 1.08
2597 reflections
170 parameters
H-atom parameters constrained
Δρ_max_ = 0.44 e Å^−3^
Δρ_min_ = −0.33 e Å^−3^

### 

Data collection: *SMART* (Bruker, 1998 ▶); cell refinement: *SAINT* (Bruker, 1999 ▶); data reduction: *SAINT* and *SHELXTL* (Sheldrick, 2008 ▶); program(s) used to solve structure: *SHELXS97* (Sheldrick, 2008 ▶); program(s) used to refine structure: *SHELXL97* (Sheldrick, 2008 ▶); molecular graphics: *SHELXTL*; software used to prepare material for publication: *SHELXTL*.

## Supplementary Material

Crystal structure: contains datablocks global, I. DOI: 10.1107/S1600536809004772/pk2146sup1.cif

Structure factors: contains datablocks I. DOI: 10.1107/S1600536809004772/pk2146Isup2.hkl

Additional supplementary materials:  crystallographic information; 3D view; checkCIF report

## Figures and Tables

**Table 1:** Hydrogen-bond geometry (Å, °)

*D*—H⋯*A*	*D*—H	H⋯*A*	*D*⋯*A*	*D*—H⋯*A*
N3—H3*B*⋯Cl1	0.86	2.29	3.144 (4)	173
N3—H3*A*⋯Cl1^i^	0.86	2.41	3.213 (4)	156
N2—H2⋯Cl2^ii^	0.86	2.21	3.029 (4)	160
N1—H1*B*⋯Cl2	0.90	2.18	3.035 (4)	159
N1—H1*A*⋯Cl1^iii^	0.90	2.20	3.076 (4)	165

Symmetry codes: (i) 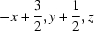; (ii) 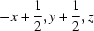; (iii) 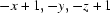.

**Fig. 1.**
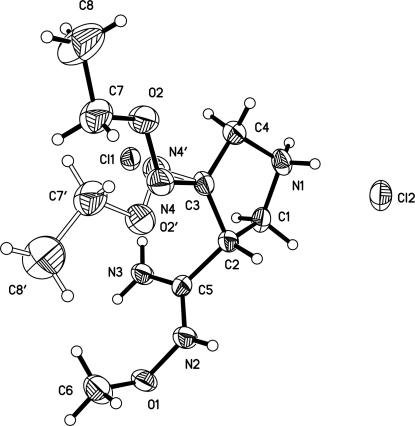
The molecular structure showing 40% probability displacement ellipsoids and the atom-numbering scheme.

**Fig. 2.**
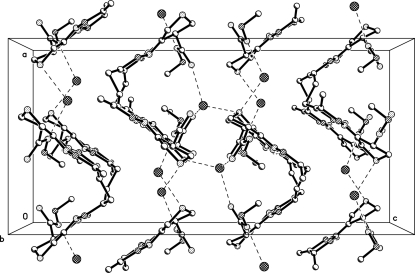
Crystal packing of the title compound viewed down the b axis.

## supplementary crystallographic information

### Comment

Since the discovery of norfloxacin, fluoroquinolone antibacterial agents have emerged as one of the dominant classes of chemotherapeutic drugs for the treatment of various bacterial infections (Ball *et al.*, 1998; Ray *et al.*, 2005). The most intensive structural variations have been carried out on the basic group at the C-7 position. In general, 5- and 6-membered nitrogen heterocycles including piperazinyl, pyrrolidinyl and piperidinyl type side chains have been proven to be the optimal substituents, as evidenced by the compounds currently on the market (Anderson & Osheroff, 2001; Choi *et al.*, 2004). Recently, as part of an ongoing program to find potent new fluoroquinolones displaying strong Gram-positive activity, we have focused our attention on introducing new functional groups to the pyrrolidine ring. We report here the crystal structure of the title compound, which is intended for use as a novel substituent at the C-7 position of fluoroquinolones.

There are two oximes in the molecule of the title compound (Fig. 1). The methyloxime has the *Z* configuration, and the ethyloxime  is disordered, with both *Z* and E configurations at occupancy factors of 0.840 (8) and 0.160 (8), respectively.  In the molecule the N3—C5(1.296 (6) Å) bond length is significantly shorter than the normal C—N single bond (1.47 Å), indicating some delocalization over the N3-C5-N2 group. The five-membered pyrrolidine ring adopts an envelope conformation. In the crystal structure, there are a number of N–H···Cl hydrogen bonds. (Table 1)

### Experimental

To a stirring solution of *N*'-methoxy-(1-*N*-*tert*-butoxycarbonyl-4- ethoxyimino) pyrrolidine-3-carboximidamide (15.0 g, 50.0 mmol) in methanol (80 ml) was pumped into dry hydrogen chloride for 2 h at room temperature. After the removal of the methanol under reduced pressure, the residue was treated with ethyl acetate (80 ml), and filtered. The filter cake was washed with ethyl acetate and ether, respectively, dried *in vacuo* to give the title compound as a white solid (11.5 g, 84.2%; mp: 375–376 K). Single crystals suitable for X-ray analysis were obtained by slow evaporation of a methanol/ ethyl acetate (1:1 *v*/*v*). ^1^H NMR(DMSO-d_6_, δ):1.14–1.17(3*H*, m, CH_3_), 3.44–3.58(m, 1*H*, pyrrolidine), 3.53(2*H*, br, NH_2_^+^), 3.66(3*H*, s, OCH_3_), 3.68–3.79(2*H*, m, OCH_2_), 4.03–4.11(4*H*, m, pyrrolidine), 9.88–9.93(3*H*, br, NH_2_, NH^+^). MS(ESI, m/*z*): 201(*M*+H)^+^.

### Refinement

All H atoms were placed at calculated positions, with C—H = 0.96–0.97 Å, N—H= 0.86–0.90 Å, and included in the final cycles of refinement using a riding model, with *U*_iso_(H) = 1.2*U*_eq_(C, N) or 1.5*U*_eq_(C) for methyl H atoms.

### Crystal data

**Table:**

C_8_H_18_N_4_O_2_^2+^·2Cl^−^	*F*(000) = 1152
*M**_r_* = 273.16	*D*_x_ = 1.223 Mg m^−^^3^
Orthorhombic, *P**b**c**n*	Mo *K*α radiation, λ = 0.71073 Å
Hall symbol: -P2n 2ab	Cell parameters from 3944 reflections
*a* = 12.7355 (14) Å	θ = 2.2–24.1°
*b* = 8.8506 (12) Å	µ = 0.43 mm^−^^1^
*c* = 26.334 (2) Å	*T* = 298 K
*V* = 2968.3 (6) Å^3^	Block, colorless
*Z* = 8	0.23 × 0.20 × 0.19 mm

### Data collection

**Table:**

Bruker SMART CCD area-detector diffractometer	2597 independent reflections
Radiation source: fine-focus sealed tube	1986 reflections with *I* > 2σ(*I*)
graphite	*R*_int_ = 0.062
φ and ω scans	θ_max_ = 25.0°, θ_min_ = 1.6°
Absorption correction: multi-scan (*SADABS*; Sheldrick, 1996)	*h* = −15→14
*T*_min_ = 0.907, *T*_max_ = 0.922	*k* = −10→10
14370 measured reflections	*l* = −29→31

### Refinement

**Table:**

Refinement on *F*^2^	Primary atom site location: structure-invariant direct methods
Least-squares matrix: full	Secondary atom site location: difference Fourier map
*R*[*F*^2^ > 2σ(*F*^2^)] = 0.077	Hydrogen site location: inferred from neighbouring sites
*wR*(*F*^2^) = 0.210	H-atom parameters constrained
*S* = 1.08	*w* = 1/[σ^2^(*F*_o_^2^) + (0.0819*P*)^2^ + 7.7771*P*] where *P* = (*F*_o_^2^ + 2*F*_c_^2^)/3
2597 reflections	(Δ/σ)_max_ = 0.001
170 parameters	Δρ_max_ = 0.44 e Å^−^^3^
0 restraints	Δρ_min_ = −0.33 e Å^−^^3^

### Special details

**Table:**

Geometry. All e.s.d.'s (except the e.s.d. in the dihedral angle between two l.s. planes) are estimated using the full covariance matrix. The cell e.s.d.'s are taken into account individually in the estimation of e.s.d.'s in distances, angles and torsion angles; correlations between e.s.d.'s in cell parameters are only used when they are defined by crystal symmetry. An approximate (isotropic) treatment of cell e.s.d.'s is used for estimating e.s.d.'s involving l.s. planes.
Refinement. Refinement of *F*^2^ against ALL reflections. The weighted *R*-factor *wR* and goodness of fit *S* are based on *F*^2^, conventional *R*-factors *R* are based on *F*, with *F* set to zero for negative *F*^2^. The threshold expression of *F*^2^ > σ(*F*^2^) is used only for calculating *R*-factors(gt) *etc*. and is not relevant to the choice of reflections for refinement. *R*-factors based on *F*^2^ are statistically about twice as large as those based on *F*, and *R*- factors based on ALL data will be even larger.

### Fractional atomic coordinates and isotropic or equivalent isotropic displacement parameters (Å^2^)

**Table:**

	*x*	*y*	*z*	*U*_iso_*/*U*_eq_	Occ. (<1)
Cl1	0.67028 (9)	0.08761 (14)	0.52171 (5)	0.0516 (4)	
Cl2	0.18814 (10)	0.22720 (17)	0.63480 (6)	0.0636 (5)	
N1	0.3711 (3)	0.1103 (5)	0.57222 (17)	0.0528 (11)	
H1A	0.3640	0.0382	0.5484	0.063*	
H1B	0.3090	0.1220	0.5881	0.063*	
N2	0.5131 (3)	0.5960 (4)	0.59228 (17)	0.0543 (11)	
H2	0.4590	0.6173	0.6105	0.065*	
N3	0.6142 (3)	0.4217 (5)	0.55190 (18)	0.0573 (12)	
H3A	0.6593	0.4897	0.5437	0.069*	
H3B	0.6239	0.3293	0.5429	0.069*	
N4	0.559 (2)	0.2442 (19)	0.6611 (11)	0.065 (4)	0.840 (8)
N4'	0.570 (12)	0.212 (14)	0.657 (6)	0.065 (4)	0.160 (8)
O1	0.5835 (3)	0.7083 (4)	0.57752 (15)	0.0579 (10)	
O2	0.5905 (4)	0.1079 (6)	0.6834 (2)	0.0762 (17)	0.840 (8)
O2'	0.581 (2)	0.352 (3)	0.6806 (10)	0.071 (8)	0.160 (8)
C1	0.4045 (4)	0.2533 (6)	0.5489 (2)	0.0503 (12)	
H1C	0.3451	0.3066	0.5343	0.060*	
H1D	0.4564	0.2359	0.5226	0.060*	
C2	0.4513 (4)	0.3417 (5)	0.59299 (19)	0.0446 (11)	
H2A	0.3944	0.3920	0.6115	0.053*	
C3	0.4953 (4)	0.2171 (5)	0.62580 (18)	0.0454 (11)	
C4	0.4533 (4)	0.0679 (6)	0.6089 (2)	0.0545 (13)	
H4A	0.5075	0.0076	0.5929	0.065*	
H4B	0.4240	0.0119	0.6372	0.065*	
C5	0.5316 (4)	0.4584 (5)	0.57781 (19)	0.0443 (11)	
C6	0.6473 (6)	0.7496 (7)	0.6196 (3)	0.0761 (18)	
H6A	0.6036	0.7852	0.6468	0.091*	
H6B	0.6949	0.8283	0.6096	0.091*	
H6C	0.6866	0.6633	0.6309	0.091*	
C7	0.6511 (8)	0.1432 (11)	0.7286 (3)	0.089 (3)	0.840 (8)
H7A	0.6104	0.2054	0.7517	0.106*	0.840 (8)
H7B	0.7149	0.1970	0.7197	0.106*	0.840 (8)
C8	0.6770 (12)	−0.0062 (15)	0.7528 (5)	0.142 (5)	0.840 (8)
H8A	0.6131	−0.0580	0.7613	0.171*	0.840 (8)
H8B	0.7174	0.0105	0.7830	0.171*	0.840 (8)
H8C	0.7169	−0.0665	0.7294	0.171*	0.840 (8)
C7'	0.648 (4)	0.337 (6)	0.7247 (18)	0.089 (3)	0.160 (8)
H7'1	0.6934	0.2488	0.7214	0.106*	0.160 (8)
H7'2	0.6072	0.3274	0.7555	0.106*	0.160 (8)
C8'	0.713 (6)	0.482 (7)	0.725 (2)	0.12 (2)	0.160 (8)
H8'1	0.7592	0.4822	0.7541	0.148*	0.160 (8)
H8'2	0.6673	0.5680	0.7270	0.148*	0.160 (8)
H8'3	0.7545	0.4884	0.6947	0.148*	0.160 (8)

### Atomic displacement parameters (Å^2^)

**Table:**

	*U*^11^	*U*^22^	*U*^33^	*U*^12^	*U*^13^	*U*^23^
Cl1	0.0413 (7)	0.0468 (7)	0.0667 (8)	0.0032 (5)	−0.0018 (6)	−0.0150 (6)
Cl2	0.0396 (7)	0.0652 (9)	0.0862 (10)	−0.0009 (6)	0.0023 (6)	−0.0207 (8)
N1	0.040 (2)	0.053 (3)	0.065 (3)	−0.010 (2)	0.003 (2)	−0.020 (2)
N2	0.049 (2)	0.039 (2)	0.074 (3)	−0.002 (2)	0.011 (2)	−0.006 (2)
N3	0.044 (2)	0.038 (2)	0.090 (3)	−0.0026 (19)	0.018 (2)	−0.007 (2)
N4	0.067 (7)	0.060 (11)	0.068 (6)	−0.008 (7)	−0.015 (5)	0.001 (8)
N4'	0.067 (7)	0.060 (11)	0.068 (6)	−0.008 (7)	−0.015 (5)	0.001 (8)
O1	0.058 (2)	0.0403 (19)	0.076 (3)	−0.0096 (17)	0.011 (2)	0.0023 (18)
O2	0.086 (4)	0.065 (3)	0.077 (3)	−0.009 (3)	−0.027 (3)	0.008 (3)
O2'	0.078 (18)	0.065 (18)	0.072 (17)	−0.006 (14)	−0.016 (14)	−0.016 (15)
C1	0.035 (2)	0.059 (3)	0.057 (3)	0.002 (2)	−0.005 (2)	−0.004 (2)
C2	0.036 (2)	0.040 (3)	0.058 (3)	−0.001 (2)	0.005 (2)	−0.002 (2)
C3	0.040 (3)	0.049 (3)	0.047 (3)	−0.009 (2)	−0.004 (2)	0.000 (2)
C4	0.049 (3)	0.046 (3)	0.068 (3)	−0.004 (2)	0.000 (3)	−0.002 (3)
C5	0.036 (2)	0.041 (3)	0.056 (3)	0.004 (2)	−0.001 (2)	−0.004 (2)
C6	0.070 (4)	0.065 (4)	0.093 (5)	−0.016 (3)	0.007 (4)	−0.014 (3)
C7	0.094 (6)	0.090 (6)	0.082 (5)	0.004 (5)	−0.034 (5)	0.001 (5)
C8	0.177 (14)	0.139 (10)	0.110 (9)	0.045 (9)	−0.059 (9)	0.010 (8)
C7'	0.094 (6)	0.090 (6)	0.082 (5)	0.004 (5)	−0.034 (5)	0.001 (5)
C8'	0.14 (5)	0.13 (5)	0.10 (4)	0.01 (4)	−0.02 (4)	−0.03 (4)

### Geometric parameters (Å, °)

**Table:**

N1—C1	1.470 (7)	C2—C3	1.509 (7)
N1—C4	1.473 (7)	C2—H2A	0.9800
N1—H1A	0.9000	C3—C4	1.493 (7)
N1—H1B	0.9000	C4—H4A	0.9700
N2—C5	1.297 (6)	C4—H4B	0.9700
N2—O1	1.393 (5)	C6—H6A	0.9600
N2—H2	0.8600	C6—H6B	0.9600
N3—C5	1.296 (6)	C6—H6C	0.9600
N3—H3A	0.8600	C7—C8	1.504 (14)
N3—H3B	0.8600	C7—H7A	0.9700
N4—C3	1.26 (3)	C7—H7B	0.9700
N4—O2	1.40 (2)	C8—H8A	0.9600
N4'—C3	1.26 (16)	C8—H8B	0.9600
N4'—O2'	1.39 (12)	C8—H8C	0.9600
O1—C6	1.423 (8)	C7'—C8'	1.53 (8)
O2—C7	1.454 (9)	C7'—H7'1	0.9700
O2'—C7'	1.45 (5)	C7'—H7'2	0.9700
C1—C2	1.521 (7)	C8'—H8'1	0.9600
C1—H1C	0.9700	C8'—H8'2	0.9600
C1—H1D	0.9700	C8'—H8'3	0.9600
C2—C5	1.507 (7)		
			
C1—N1—C4	106.7 (4)	N1—C4—H4B	111.2
C1—N1—H1A	110.4	C3—C4—H4B	111.2
C4—N1—H1A	110.4	H4A—C4—H4B	109.1
C1—N1—H1B	110.4	N3—C5—N2	122.5 (5)
C4—N1—H1B	110.4	N3—C5—C2	121.2 (4)
H1A—N1—H1B	108.6	N2—C5—C2	116.3 (4)
C5—N2—O1	118.1 (4)	O1—C6—H6A	109.5
C5—N2—H2	120.9	O1—C6—H6B	109.5
O1—N2—H2	120.9	H6A—C6—H6B	109.5
C5—N3—H3A	120.0	O1—C6—H6C	109.5
C5—N3—H3B	120.0	H6A—C6—H6C	109.5
H3A—N3—H3B	120.0	H6B—C6—H6C	109.5
C3—N4—O2	109.2 (14)	O2—C7—C8	105.9 (8)
C3—N4'—O2'	109 (9)	O2—C7—H7A	110.6
N2—O1—C6	109.5 (4)	C8—C7—H7A	110.6
N4—O2—C7	108.0 (11)	O2—C7—H7B	110.6
N4'—O2'—C7'	109 (7)	C8—C7—H7B	110.6
N1—C1—C2	103.8 (4)	H7A—C7—H7B	108.7
N1—C1—H1C	111.0	C7—C8—H8A	109.5
C2—C1—H1C	111.0	C7—C8—H8B	109.5
N1—C1—H1D	111.0	H8A—C8—H8B	109.5
C2—C1—H1D	111.0	C7—C8—H8C	109.5
H1C—C1—H1D	109.0	H8A—C8—H8C	109.5
C5—C2—C3	113.7 (4)	H8B—C8—H8C	109.5
C5—C2—C1	114.6 (4)	O2'—C7'—C8'	104 (4)
C3—C2—C1	101.9 (4)	O2'—C7'—H7'1	110.9
C5—C2—H2A	108.8	C8'—C7'—H7'1	110.9
C3—C2—H2A	108.8	O2'—C7'—H7'2	110.9
C1—C2—H2A	108.8	C8'—C7'—H7'2	110.9
N4—C3—C4	128.4 (10)	H7'1—C7'—H7'2	108.9
N4'—C3—C4	116 (6)	C7'—C8'—H8'1	109.5
N4—C3—C2	121.6 (10)	C7'—C8'—H8'2	109.5
N4'—C3—C2	133 (7)	H8'1—C8'—H8'2	109.5
C4—C3—C2	110.0 (4)	C7'—C8'—H8'3	109.5
N1—C4—C3	103.0 (4)	H8'1—C8'—H8'3	109.5
N1—C4—H4A	111.2	H8'2—C8'—H8'3	109.5
C3—C4—H4A	111.2		
			
C5—N2—O1—C6	−104.6 (6)	C5—C2—C3—C4	−137.4 (4)
C3—N4—O2—C7	−171.4 (14)	C1—C2—C3—C4	−13.5 (5)
C3—N4'—O2'—C7'	167 (9)	C1—N1—C4—C3	30.2 (5)
C4—N1—C1—C2	−39.6 (5)	N4—C3—C4—N1	171.6 (16)
N1—C1—C2—C5	154.7 (4)	N4'—C3—C4—N1	−179 (8)
N1—C1—C2—C3	31.5 (5)	C2—C3—C4—N1	−9.5 (5)
O2—N4—C3—C4	1(3)	O1—N2—C5—N3	2.4 (8)
O2—N4—C3—C2	−177.3 (9)	O1—N2—C5—C2	−177.8 (4)
O2'—N4'—C3—N4	−13 (25)	C3—C2—C5—N3	60.0 (6)
O2'—N4'—C3—C4	−163 (7)	C1—C2—C5—N3	−56.7 (6)
O2'—N4'—C3—C2	31 (17)	C3—C2—C5—N2	−119.7 (5)
C5—C2—C3—N4	41.6 (15)	C1—C2—C5—N2	123.6 (5)
C1—C2—C3—N4	165.4 (14)	N4—O2—C7—C8	176.7 (14)
C5—C2—C3—N4'	29 (9)	N4'—O2'—C7'—C8'	143 (8)
C1—C2—C3—N4'	153 (9)		

### Hydrogen-bond geometry (Å, °)

**Table:**

*D*—H···*A*	*D*—H	H···*A*	*D*···*A*	*D*—H···*A*
N3—H3B···Cl1	0.86	2.29	3.144 (4)	173
N3—H3A···Cl1^i^	0.86	2.41	3.213 (4)	156
N2—H2···Cl2^ii^	0.86	2.21	3.029 (4)	160
N1—H1B···Cl2	0.90	2.18	3.035 (4)	159
N1—H1A···Cl1^iii^	0.90	2.20	3.076 (4)	165

Symmetry codes: (i) −*x*+3/2, *y*+1/2, *z*; (ii) −*x*+1/2, *y*+1/2, *z*; (iii) −*x*+1, −*y*, −*z*+1.
